# Predictivity of Hepatic Steatosis Index for Gestational Hypertension and Preeclampsia: a Prospective Cohort Study

**DOI:** 10.7150/ijms.104943

**Published:** 2025-01-21

**Authors:** Lin Zhang, Shen Gao, Yingyi Luan, Shaofei Su, Enjie Zhang, Jianhui Liu, Shuanghua Xie, Yue Zhang, Wentao Yue, Ruixia Liu, Chenghong Yin

**Affiliations:** 1 Department of Internal Medicine, Beijing Obstetrics and Gynecology Hospital, Capital Medical University, Beijing Maternal and Child Health Care Hospital, China.; 2 Department of Central Laboratory, Beijing Obstetrics and Gynecology Hospital, Capital Medical University, Beijing Maternal and Child Health Care Hospital, China.; 3 Department of Research Management, Beijing Obstetrics and Gynecology Hospital, Capital Medical University, Beijing Maternal and Child Health Care Hospital, China.

**Keywords:** hepatic steatosis index, non-alcoholic fatty liver disease, gestational hypertension, preeclampsia

## Abstract

**Context:** Previous studies have reported that pregnant women with non-alcoholic fatty liver disease (NAFLD) face an increased risk of gestational hypertension (GH) and preeclampsia (PE). However, no study has assessed the relationship between the Hepatic Steatosis Index (HSI), a biomarker for NAFLD, in early pregnancy and the subsequent risk of GH and PE.

**Objective:** We aimed to investigate the relationship between HSI in early pregnancy and the risks of GH and PE in Chinese women.

**Methods:** Based on the China Birth Cohort Study conducted from February 2018 to December 2022, this prospective cohort study collected liver enzyme and body mass index data from pregnant participants during 6-13^+6^ gestational weeks. The incidences of GH and PE were monitored until delivery.

**Results:** This study included 39,114 pregnant women, and GH and PE incidences were 4.2% and 4.1%, respectively. After multivariable adjustment, the risks of GH (Q2: OR = 1.35, 95% CI = 1.13-1.62; Q3: OR = 1.86, 95% CI = 1.57-2.20; Q4: OR = 3.81, 95% CI = 3.25-4.46) and PE (Q2: OR = 1.22, 95% CI = 1.01-1.47; Q3: OR = 1.96, 95% CI = 1.65-2.32; Q4: OR = 3.60, 95% CI = 3.07-4.22) significantly increased with higher HSI quartiles. Further analysis indicated that compared to women aged 35 years or older, HSI in pregnant women under 35 years had relatively stronger predictive value for GH (OR ≥ 35 = 4.527, 95% CI = 3.762-5.446 vs. OR < 35 = 2.325, 95% CI = 1.729-3.128) and PE (OR ≥ 35 = 4.13, 95% CI = 3.433-4.983 vs. OR < 35 = 2.348, 95% CI = 1.736-3.176).

**Conclusion**: Elevated HSI may be associated with an increased risk of GH and PE.

## 1. Introduction

Preeclampsia (PE), a pregnancy-specific disease that manifests after 20 weeks of gestation, is characterized by hypertension and proteinuria. It can lead to various organ dysfunctions, such as liver damage, and cardiac and renal insufficiency, posing significant risks to both mother and fetus 1, 2. Gestational hypertension (GH) is defined as elevated blood pressure alone, occurring after 20 weeks of gestation without accompanying organic dysfunction 3.

Non-alcoholic fatty liver disease (NAFLD), a leading cause of liver diseases globally 4, is characterized by excessive hepatic fat accumulation and insulin resistance and is defined as the histological presence of steatosis in more than 5% of hepatocytes 5. Meta-analyses estimate that the prevalence of NAFLD is about 25%, having nearly tripled over the past decade 6, 7.

Established clinical risk factors for PE include hypertension, diabetes, obesity, advanced maternal age (> 35 years), obstructive sleep apnea syndrome, kidney disease, thrombophilia, primiparity, and assisted reproduction 3, 8. Previous studies have reported that the combined risk of GH and PE is nearly fourfold higher in pregnant women with NAFLD 7, 9. Thus, the inclusion of NAFLD in predictive risk stratification for PE is gaining recognition 10. Compared to traditional ultrasound for screening NAFLD, the Hepatic Steatosis Index (HSI) is a more efficient, cost-effective, and equally accurate biomarker derived from body mass index (BMI) and liver enzyme calculations. An HSI value of 36.0 detects NAFLD with a specificity of 93.1% (95% CI, 92.0-94.0) and a positive likelihood ratio of 6.505 (95% CI, 5.628-7.519) 11, 12. However, data on the relationship between HSI and GH and HSI and PE remain limited.

We collected data from a large prospective cohort study in China to evaluate the predictive value of HSI for GH and PE, aiming to demonstrate the increased risk of GH and PE associated with NAFLD.

## 2. Materials and Methods

### Study population

This study enrolled participants from the China Birth Cohort Study (CBCS) between February 2018 and December 2022 who delivered a live-born singleton at Beijing Obstetrics and Gynecology Hospital, Capital Medical University. Participants were excluded for the following reasons: (1) incomplete liver enzyme tests in the first trimester, (2) a diagnosis of hypertension before pregnancy, (3) severe renal diseases, chronic hepatitis, autoimmune disorders, or related conditions, and (4) missing covariate data. All participants completed baseline questionnaires and clinical laboratory tests at recruitment (6-13^+6^ gestational weeks) and continued regular follow-ups until delivery 13. The final study population included 39,114 pregnant women. Figure S1 shows the participant selection process. This study was approved by the Ethics Committee of Beijing Obstetrics and Gynecology Hospital, Capital Medical University (approval number: 2018-KY-003-02). All study participants provided written informed consent.

### Exposure and covariates

Demographic and obstetric characteristics of study participants, such as maternal age, ethnicity, employment status, educational level, pre-pregnancy height and weight, maternal lifestyle factors (smoking status, alcohol consumption, and folic acid supplementation), gravidity, mode of fertilization, obstetric history, and medical history, were obtained at baseline using a standardized questionnaire. The date of the last menstrual period was confirmed through ultrasound during the first trimester. Pre-pregnancy BMI was calculated as maternal weight (kg)/height squared (m^2^) and classified into normal (< 24.0 kg/m^2^), overweight (24.0-27.9 kg/m^2^), and obese (≥ 28.0 kg/m^2^) categories, based on established thresholds 14. Laboratory test results, including fasting blood glucose (FBG), triglyceride (TG), total cholesterol (TC), low-density lipoprotein cholesterol (LDL-C), high-density lipoprotein cholesterol (HDL-C), aspartate aminotransferase (AST), alanine aminotransferase (ALT), and γ-glutamyl transferase (GGT), were obtained after approximately 12 hours of fasting during 6-13^+6^ weeks of gestation and were collected from the medical records. HSI was calculated as (8 × ALT/AST) + BMI + 2 12.

### Definition of GH and PE

The occurrence of GH and PE was recorded during follow-up and verified by the diagnoses in the medical records after delivery. GH and PE were diagnosed according to the guidelines recommended by the International Society for the Study of Hypertension in Pregnancy (ISSHP) 8. PE is defined as gestational hypertension accompanied by proteinuria and/or other maternal organ dysfunction at or after 20 weeks of gestation. In contrast, GH is persistent de novo hypertension developing at or after 20 weeks of gestation without features of PE.

### Statistical analyses

Participants were classified into three groups: normal, GH, and PE. Baseline characteristics were reported as mean ± standard deviation (SD) or median (interquartile range) for continuous variables. One-way analysis of variance (ANOVA) or Kruskal-Wallis test was used to compare the differences across the normal, GH, and PE groups. Categorical variables were expressed as frequency (%) and compared across groups using the χ^2^ test. Logistic regression models were used to calculate odds ratios (ORs) and 95% confidence intervals (CIs) for liver enzyme levels (AST, ALT, GGT) and HSI quartiles in relation to GH and PE, adjusting for potential covariates such as gestational age of recruitment, maternal age, pre-pregnancy BMI, folic acid supplementation, primiparity, FBG, and TC. Subgroup analyses were performed by stratifying participants according to maternal age (< 35 years or ≥ 35 years), first-trimester FBG (< 5.6 or ≥ 5.6 mmol/L), and gravidity (> 1 or 1). ORs between subgroups were compared using the z-statistic 15. Receiver Operating Characteristic Curve (ROC) analysis was used to evaluate the predictive capacity of liver enzyme levels and HSI for GH and PE. Restricted cubic spline (RCS) was applied to develop two non-linear regression models for GH and PE, with four knots positioned at the 5th, 35th, 65th, and 95th percentiles of predictor distributions. Statistical significance was defined as *P* < 0.05 (two-sided). All statistical analyses were performed using SAS version 9.4 (SAS Institute Inc., Cary, NC, USA).

## 3. Results

### Clinical features of the enrolled pregnant women

Among the 39,114 participants who had an average age of 32.0 years and were at 8 gestational weeks at baseline, 1,668 (4.3%) and 1,588 (4.1%) were diagnosed with GH and PE, respectively. The baseline characteristics of the study participants are shown in Table 1. Participants in the normal group had higher proportions of individuals with higher education levels and natural pregnancies (*P*<0.001). Furthermore, participants in the GH or PE groups were more likely to have a higher pre-pregnancy BMI or to be experiencing their first pregnancy (*P* < 0.001). The overall median levels of the liver enzyme indicators—AST, ALT, and GGT—were 14.8 (13.0-17.4) U/L, 12.9 (9.9-18.8) U/L, and 13.0 (11.0-18.0) U/L, respectively. The mean HSI was 31.7 ± 5.2. All serum markers, including liver enzyme indicators, HSI, FBG, and blood lipid indicators (TG, TC, LDL-C, and HDL-C) in the first trimester, showed significant differences among the normal, GH, and PE groups (*P* < 0.001).

### Associations of liver enzyme levels in the first trimester with risk of GH and PE

The associations between AST, ALT, GGT, HSI, and the risks of GH and PE are shown in Table 2. After adjusting for confounding variables, the risk of GH was significantly higher among participants in the third and fourth quartiles of ALT and GGT (ALT Q3: OR = 1.248, 95% CI: 1.070-1.455, Q4: OR = 1.610, 95% CI: 1.390-1.865; GGT Q3: OR = 1.410, 95% CI: 1.201-1.655, Q4: OR = 1.847, 95% CI: 1.570-2.172) compared with those in the first quartile. Moreover, the risk of GH increased significantly with higher HSI quartiles compared with the first quartile (Q2: OR = 1.354, 95% CI: 1.131-1.621; Q3: OR = 1.858, 95% CI: 1.567-2.203; Q4: OR = 3.808, 95% CI: 3.254-4.457). Similarly, PE risk increased with higher liver enzyme quartiles and HSI. In fully adjusted models, the risk of PE was significantly higher among participants in the highest quartile of AST (OR = 1.256, 95% CI: 1.090-1.447) and the third and fourth quartiles of ALT (Q3: OR = 1.255, 95% CI: 1.075-1.464, Q4: OR = 1.538, 95% CI: 1.325-1.785). Furthermore, the risk of PE increased significantly with higher quartiles of GGT and HSI (GGT Q2: OR = 1.274, 95% CI: 1.055-1.537; Q3: OR = 1.467, 95% CI: 1.239-1.737; Q4: OR = 1.978, 95% CI: 1.669-2.344; HSI Q2: OR = 1.220, 95% CI: 1.014-1.468; Q3: OR = 1.957, 95% CI: 1.651-2.321; Q4: OR = 3.602, 95% CI: 3.072-4.224).

The results of subgroup analyses are shown in Table 3. The relationship between ALT, GGT, and HSI with the risk of GH was stronger among pregnant women under 35 years of age. Similarly, the risk of PE associated with HSI was higher in pregnant women younger than 35 years. There was no significant difference in the association between the top quartiles of AST, ALT, GGT, and HSI and GH or PE across subgroups stratified by baseline FBG levels and gravidity (All *P* > 0.05).

**Figure 1:**
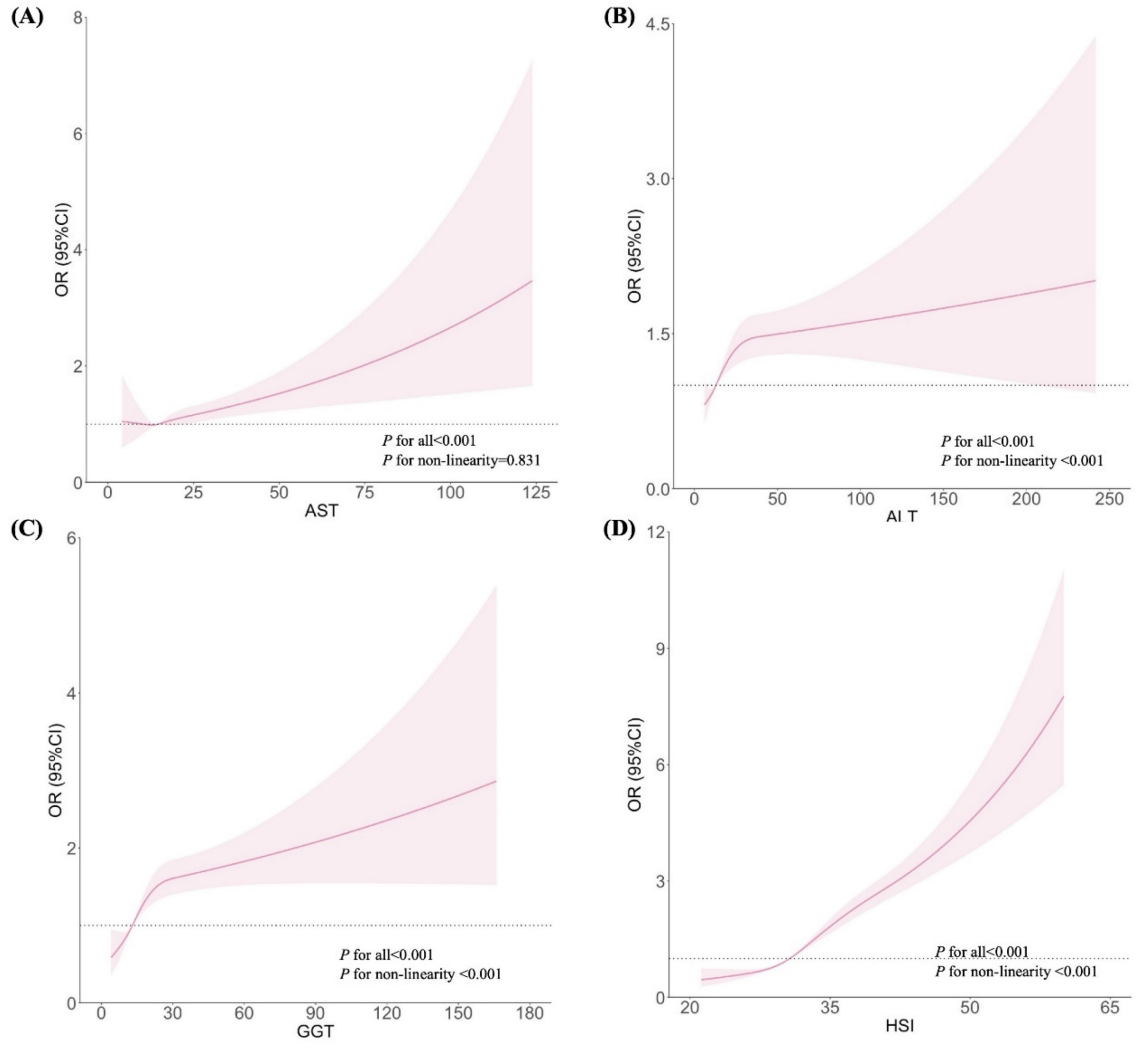
Associations between liver enzyme indicators and GH, plotted with restricted cubic splines. Odds ratios and 95% CI were estimated using logistic regression model adjusted for the maternal age, pre-pregnancy BMI (excepted for HSI), FA supplement, first pregnancy, FBG, TC and gestational week.

**Figure 2:**
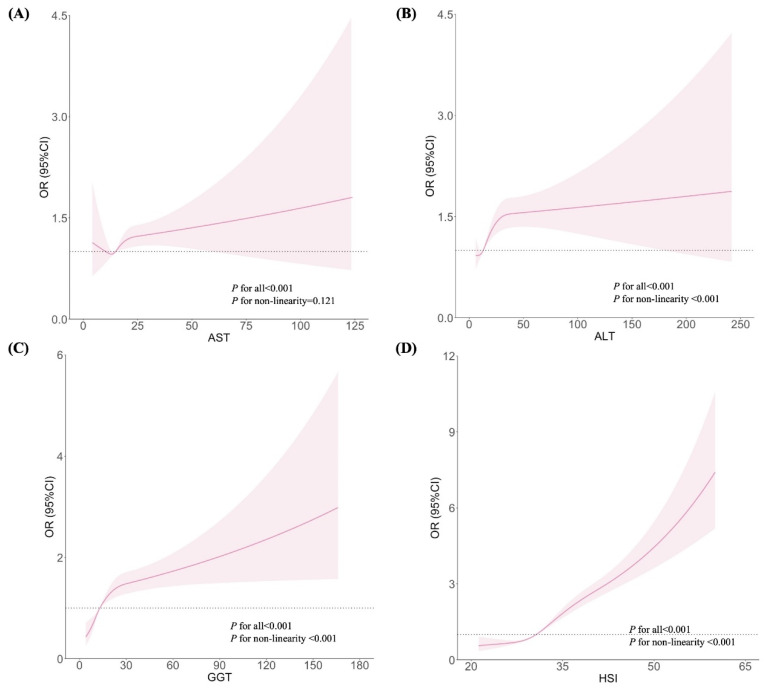
Associations between liver enzyme indicators and PE, plotted with restricted cubic splines. Odds ratios and 95% CI were estimated using logistic regression model adjusted for the maternal age, pre-pregnancy BMI (excepted for HSI), FA supplement, first pregnancy, FBG, TC and gestational week.

Figures 1 and 2 show the dose-response relationships between AST, ALT, GGT, and HSI with the risks of GH and PE, respectively. After adjustment for confounders, non-linear relationships were demonstrated between ALT, GGT, and HSI and the risk of GH (Figures 1B, 1C, and 1D; all *P* for non-linearity < 0.001). Similar non-linear associations were observed between ALT, GGT, HSI and the risk of PE (Figures 2B, 2C, and 2D; all *P* for non-linearity < 0.001). Maternal AST levels showed a linear association with the risks of GH (Figure 1A; *P* < 0.001,* P* for non-linearity = 0.831) and PE (Figure 2A;* P* < 0.001,* P* for non-linearity = 0.121).

**Figure 3:**
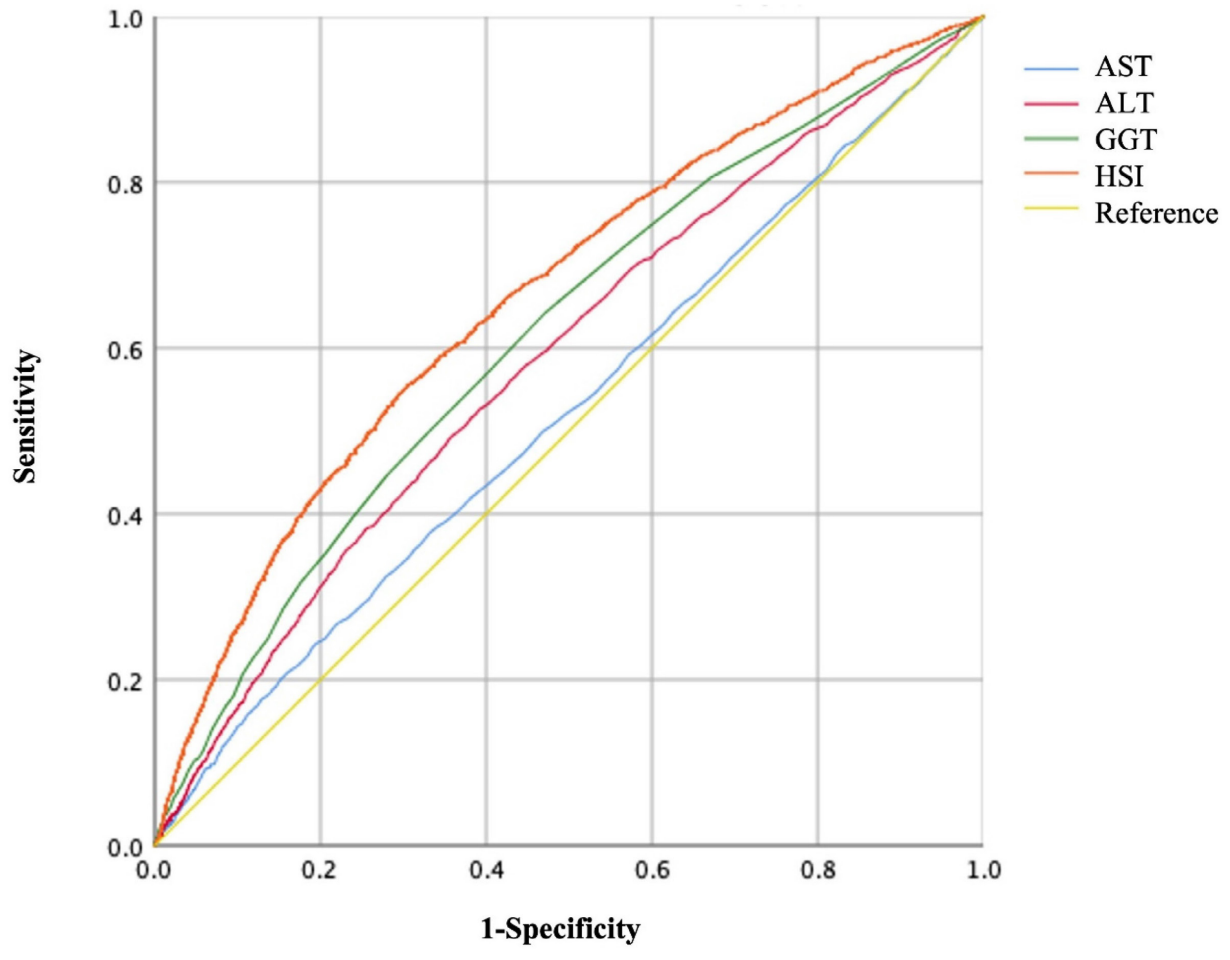
Receiver operating characteristic (ROC) curves of liver enzyme indicators to predict GH.

**Figure 4:**
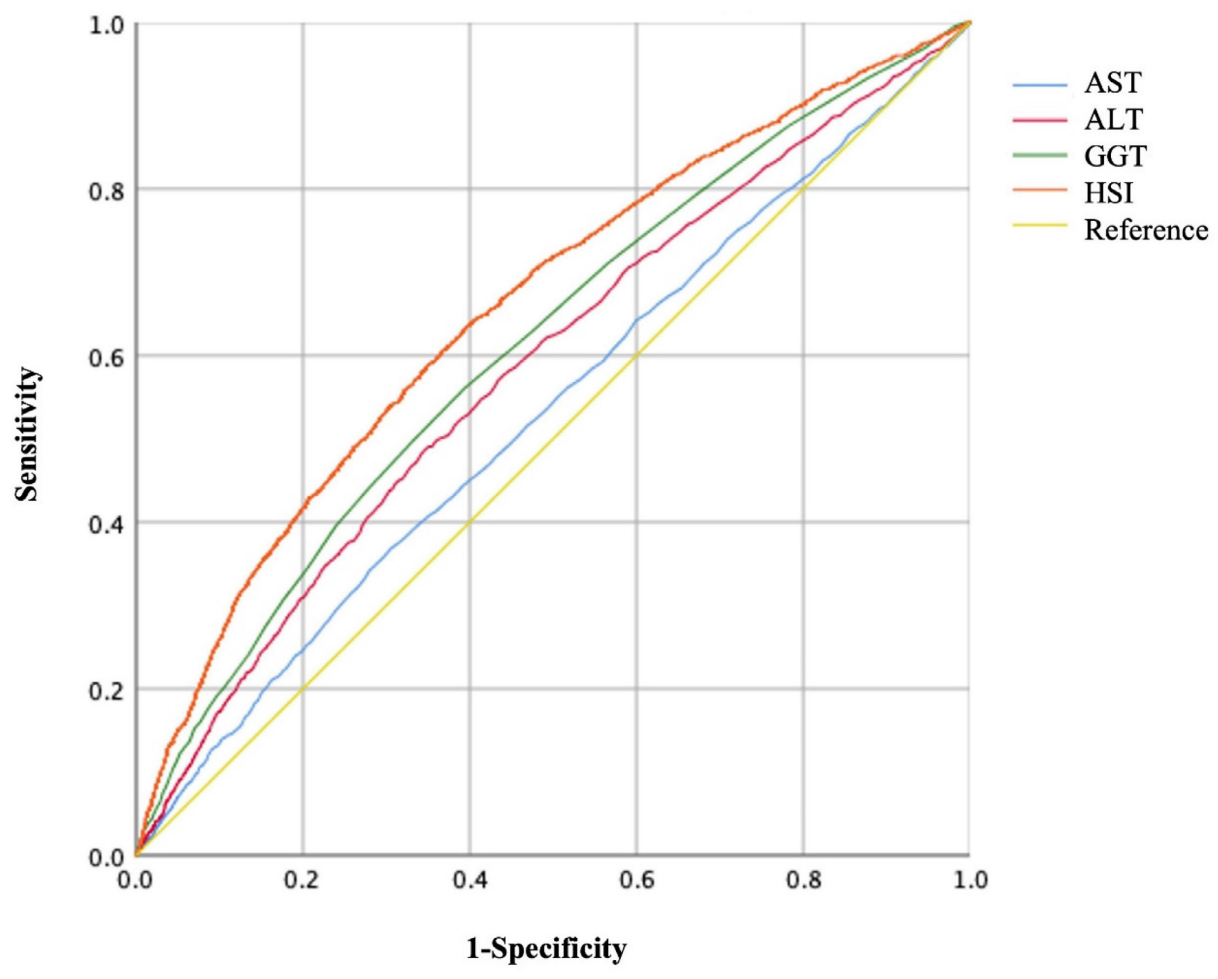
Receiver operating characteristic (ROC) curves of liver enzyme indicators to predict PE.

The areas under the ROC curves (AUC) to predict the risks of GH and PE using AST, ALT, GGT, and HSI are shown in Figures 3, 4, and Table 5. Maternal HSI in the first trimester showed the best predictive performance for GH and PE, yielding AUCs of 0.663 (95% CI: 0.649-0.676) and 0.657 (95% CI: 0.643-0.671), respectively, which were significantly higher than those for AST, ALT, and GGT (all *P* < 0.001).

## 4. Discussion

The results indicated that the proportion of subjects with higher education levels and natural pregnancies was greater in the normal group (*P* < 0.001). Furthermore, participants in the GH or PE groups were more likely to have higher pre-pregnancy BMI or be experiencing their first pregnancy (*P* < 0.001). All serum markers, including liver enzyme indicators, HSI, FBG, and blood lipid indicators (TG, TC, LDL-C, and HDL-C) in the first trimester, showed significant differences among the normal, GH, and PE groups (*P* < 0.001). In our cohort study, the incidence of GH and PE gradually increased with higher HSI. After model calibration, women in the highest quartile of HSI had a threefold increase in the odds of GH compared to those in the lowest quartile. The risk of PE significantly increased with higher HSI quartiles, with a threefold increase in the odds of PE in the highest quartile. Pregnant women under the age of 35 years also exhibited a stronger relationship between HSI and GH and between HSI and PE compared to women over 35 years.

In our study population, the rates of developing GH and PE were 4.3% and 4.1% among pregnant women who delivered a live-born singleton, similar to previous reports 2, 16. Our findings align with prior research indicating that blood glucose levels, primiparity, assisted reproduction, pre-pregnancy BMI, and advanced maternal age all positively correlate with the risks of GH and PE 3, 8. Additionally, we found that pregnant women in the GH and PE groups had higher levels of TC, TG, LDL-C, and HDL-C than the normal group. Since atherogenic dyslipidemia is a known proximate cause of endothelial dysfunction, this association may help explain our findings 17.

Based on the results of the present study, after adjusting for confounders, non-linear relationships were demonstrated between ALT, GGT, HSI and the risk of GH (all *P* for non-linearity < 0.001). Similar non-linear associations were found between ALT, GGT, HSI, and the risk of PE (all *P* for non-linearity < 0.001). Maternal AST levels were linearly associated with the risk of both GH (*P* < 0.001) and PE (*P* < 0.001). Several studies support our results. A Korean study involving 2,322 women revealed that those with elevated ALT levels in the first trimester had three times the odds of developing PE (OR = 3.24, 95% CI: 1.06-8.41) 18. A prospective cohort study from China involving 5,685 pregnant women found that, after adjusting for potential covariates, high-normal levels of ALT and GGT were associated with an increased risk of GH (OR_ALT_ = 1.21, 95% CI: 1.05-1.38; OR_GGT_ = 1.23, 95% CI: 1.09-1.39) and PE (OR_ALT_ = 1.15, 95% CI: 1.03-1.28; OR_GGT_ = 1.28, 95% CI: 1.16-1.41), respectively 19. Moreover, a prospective cohort study of 1,041 women in South China indicated that elevated GGT levels increased the risk of PE and GH in early pregnancy by 2.6 times (OR = 2.61, 95% CI: 1.05-6.83) 20. In all three studies, pregnant women with known chronic viral hepatitis B, hepatitis C, or other liver diseases were excluded. The authors also imposed that unknown elevated liver enzyme levels may indicate pre-existing NAFLD 18 19.

However, previous studies have reported no correlation between AST levels and the risk of PE. In our study, AST was positively correlated with an increased risk of PE, showing a 1.2-fold higher risk in the highest quartile of HSI compared to the lowest among all pregnant participants. Similar conclusions were reported by Elad Mei-Dan *et al.* and A Bülez *et al*. 21, 22. It is well known that AST plays a role in amino acid metabolism and the tricarboxylic acid cycle 23. We suggest that AST may affect the epithelial-mesenchymal transition 24 by inhibiting the Phospho-Akt signaling pathway 25 in patients with PE.

HSI is a non-invasive biomarker used to diagnose NAFLD and has demonstrated good performance in detecting the presence of hepatic steatosis in both clinical and epidemiological studies 26, 12, 27-30. Our study proposes that HSI may serve as a risk factor for GH and PE. Several studies have similarly supported our conclusions. Monika Sarkar *et al.* demonstrated that, after controlling for confounding factors, pregnant women with NAFLD exhibited an elevated risk of PE, including HELLP syndrome (Hemolysis, Elevated Liver enzymes, Low platelets) and eclampsia (OR = 3.1, 95% CI = 2.6-3.8), when compared to those without chronic liver disease 7. Hydar *et al.* conducted a meta-analysis of 13,641 participants and found that pregnant women with NAFLD had a significantly higher likelihood of GH (OR = 1.83; 95% CI = 1.03-3.26; *P* = 0.041; n = 2) and PE (OR = 2.43; 95% CI = 1.46-4.04; *P* = 0.001; n = 3). A meta-analysis reported that the incidence of the composite outcome of PE, eclampsia, or HELLP syndrome in pregnancies with NAFLD was almost four times higher than in the control group (OR = 3.91, 95% CI = 2.71-5.64, n = 4) 9. A retrospective study in China involving 14,708 pregnant women revealed that, after adjusting for potential confounding factors, NAFLD significantly elevated the risk of GH (OR = 3.054, 95% CI = 2.191-4.257) and PE/eclampsia (OR = 3.994, 95% CI = 2.591-6.005) 31.

Our study demonstrated that, compared to pregnant women aged 35 years or older, HSI exhibited stronger predictive power in those under the age of 35 years for GH (OR_≥35_ = 4.527, 95% CI = 3.762-5.446 vs. OR_<35_ = 2.325, 95% CI = 1.729-3.128) and PE (OR_≥35_ = 4.13, 95% CI = 3.433-4.983 vs. OR_<35_ = 2.348, 95% CI = 1.736-3.176). We suggest that younger patients might be more susceptible to the effects of NAFLD. For example, Walker RW *et al*. reported that the same variant in the PNPLA3 gene played different roles depending on the age of NAFLD onset, suggesting that age may play a crucial role in the development of NAFLD 32. This finding may explain the varying effects of age on GH and PE.

NAFLD could lead to hepatic and peripheral insulin resistance and exacerbate liver inflammation 33. NAFLD frequently coexists with obesity, diabetes, dyslipidemia, hypertension, and other metabolic disorders 34 and is increasingly recognized as a key feature and liver-specific manifestation of metabolic syndrome 35.

The mechanism by which NAFLD affects GH and PE during pregnancy may involve pregnancy-induced insulin resistance, a normal adaptation to ensure adequate carbohydrate supply for fetal growth 36. NAFLD during pregnancy intensifies this state of insulin resistance 37. Vascular insulin resistance promotes vascular contraction by impairing the phosphatidylinositol 3-kinase pathway and subsequently reducing endothelial nitric oxide (NO) production, thereby promoting the onset of PE 38-40. Furthermore, NAFLD induces a systemic inflammatory response, demonstrated by elevated inflammatory factors, including Interleukin 6 (IL-6) Tumour necrosis factor α(TNF-α), and C-C motif ligand 2(CCL2) 41, 42. This inflammation leads to endothelial dysfunction 43, contributing to the development of PE 44.

While invasive liver biopsy is the gold standard for diagnosing NAFLD 45, its large-scale use is challenging. Magnetic resonance imaging is time-consuming and expensive 46, whereas ultrasound exhibits low sensitivity and depends on operator skill 47. Consequently, HSI represents a suitable biomarker for NAFLD screening.

This study has several strengths. First, we found that elevated HSI in early pregnancy predicts an increased risk for GH and PE. To our knowledge, this is the first large-scale cohort study linking pregnancy HSI with the risk of GH and PE, providing new evidence of the impact of NAFLD on these conditions. This discovery bears direct significance for pregnancy counseling for women with NAFLD. Second, we found that HSI predicts GH and PE more strongly in pregnant women under 35. Given the potential role of genetic factors in the onset age of NAFLD, our findings offer new clinical evidence to guide future research on genetic influences in NAFLD.

Our study had some limitations. First, this single-center observational study is subject to potential selection and information biases despite the large sample size. Second, similar to most studies, we could not perform tissue biopsies to confirm the presence of NAFLD in these participants 5. Third, this study included only pregnant women with live singletons, suggesting that further research should consider including cases of pregnancy loss.

## 5. Conclusion

In conclusion, our study showed that elevated HSI in the first trimester was significantly associated with an increased risk of GH and PE in a large sample of Chinese pregnant women. Notably, higher levels of AST in early pregnancy were also positively correlated with an increased risk of PE. Moreover, our results suggest that HSI in pregnant women under 35 had a stronger predictive value for GH and PE. These findings illustrate the importance of NAFLD as a potential risk factor for GH and PE and highlight the need for in-depth research into perinatal primary prevention measures for women with NAFLD.

## Supplementary Material

Supplementary figure 1: Flow chart.

## Figures and Tables

**Table 1:** Baseline characteristics of study subjects.

Variables	Total (N=39114)	Normal (N=35858)	GH (N=1668)	PE (N=1588)	*P*-value
Maternal age, year	32.0±3.8	32.0±3.8	32.5±4.1	32.4±4.2	<0.001
Maternal employed	36451 (93.2)	33437 (93.2)	1544 (92.6)	1470 (92.6)	0.336
Gestational week, weeks	8.0 (7.0-9.0)	8.0 (7.0-9.0)	8.0 (7.0-9.0)	8.0 (7.0-9.0)	0.011
Han ethics	36194 (92.5)	33189 (92.6)	1539 (92.3)	1466 (92.3)	0.857
Maternal education					<0.001
< College	2273 (5.8)	2042 (5.7)	113 (6.8)	118 (7.4)	
Undergraduate/College	26963 (68.9)	24599 (68.6)	1199 (71.9)	1165 (73.4)	
> Postgraduate or higher	9878 (25.3)	9217 (25.7)	356 (21.3)	305 (19.2)	
Pre-pregnancy BMI, kg/m^2^					<0.001
< 24	31309 (80.0)	29361 (81.9)	995 (59.7)	953 (60.0)	
24-28	6130 (15.7)	5237 (14.6)	448 (26.9)	445 (28.0)	
≥ 28	1675 (4.3)	1260 (3.5)	225 (13.5)	190 (12.0)	
FA supplement	34982 (89.4)	32071 (89.4)	1490 (89.3)	1421 (89.5)	0.989
Alcohol use during the first trimester	1810 (4.6)	1670 (4.7)	71 (4.3)	69 (4.3)	0.644
Maternal smoking during the first trimester	86 (0.2)	78 (0.2)	4 (0.2)	4 (0.3)	0.945
First pregnancy	21137 (54.0)	19251 (53.7)	938 (56.2)	948 (59.7)	<0.001
Natural pregnancy	36724 (93.9)	33807 (94.3)	1503 (90.1)	1414 (89.0)	<0.001
Serum markers during the first trimester					
AST, U/L	14.8 (13.0-17.4)	14.8 (12.9-17.4)	15.0 (13.0-18.1)	15.2 (13.2-18.2)	<0.001
ALT, U/L	12.9 (9.9-18.8)	12.8 (9.8-18.4)	15.1 (10.9-23.4)	15.2 (10.8-23.3)	<0.001
GGT, U/L	13.0 (11.0-18.0)	13.0 (11.0-17.0)	15.0 (12.0-21.0)	15.0 (12.0-21.0)	<0.001
HSI	31.7±5.2	31.5±5.0	34.6±6.0	34.5±6.1	<0.001
FBG, mmol/L	4.6±0.4	4.6±0.4	4.7±0.4	4.7±0.4	<0.001
TG, mmol/L	1.0 (0.8-1.3)	1.0 (0.8-1.2)	1.1 (0.8-1.5)	1.1 (0.9-1.5)	<0.001
TC, mmol/L	4.2±0.7	4.2±0.7	4.3±0.7	4.4±0.7	<0.001
LDL-C, mmol/L	2.2±0.6	2.2±0.6	2.4±0.6	2.4±0.6	<0.001
HDL-C, mmol/L	1.5±0.3	1.5±0.3	1.5±0.3	1.5±0.3	<0.001

Abbreviations: GH, gestational hypertension; PE, preeclampsia; BMI, body mass index; FA, folic acid; FBG, fasting blood glucose; TG, triglyceride; TC, total cholesterol; LDL-C, low-density lipoprotein cholesterol; HDL-C, high-density lipoprotein cholesterol; AST, aspartate aminotransferase; ALT, alanine aminotransferase; GGT, γ-glutamyl transferase; HSI, hepatic steatosis index.

**Table 2:** Associations of liver enzyme levels in the first trimester with risk of GH and PE.

Variables	GH		PE	
	Model 1	Model 2^*^	Model 1	Model 2^*^
AST, U/L				
Q1 (<13.00)	1.000	1.000	1.000	1.000
Q2 (13.00-14.79)	0.990 (0.858-1.142)	1.025 (0.888-1.185)	1.017 (0.876-1.181)	1.041 (0.895-1.211)
Q3 (14.80-17.39)	0.964 (0.836-1.111)	1.002 (0.868-1.157)	1.060 (0.915-1.227)	1.090 (0.940-1.264)
Q4 (≥17.40)	1.206 (1.053-1.381)	1.131 (0.985-1.298)	1.356 (1.179-1.559)	1.256 (1.090-1.447)
ALT, U/L				
Q1 (<9.90)	1.000	1.000	1.000	1.000
Q2 (9.90-12.89)	1.186 (1.013-1.39)	1.104 (0.941-1.294)	1.078 (0.916-1.268)	1.009 (0.856-1.188)
Q3 (12.90-18.79)	1.441 (1.238-1.677)	1.248 (1.070-1.455)	1.443 (1.239-1.682)	1.255 (1.075-1.464)
Q4 (≥18.80)	2.187 (1.897-2.522)	1.610 (1.390-1.865)	2.090 (1.809-2.414)	1.538 (1.325-1.785)
GGT, U/L				
Q1 (<11.00)	1.000	1.000	1.000	1.000
Q2 (11.00-12.99)	1.100 (0.914-1.324)	1.047 (0.869-1.261)	1.331 (1.103-1.605)	1.274 (1.055-1.537)
Q3 (13.00-17.99)	1.633 (1.394-1.914)	1.410 (1.201-1.655)	1.690 (1.430-1.997)	1.467 (1.239-1.737)
Q4 (≥18.00)	2.713 (2.323-3.168)	1.847 (1.570-2.172)	2.890 (2.456-3.400)	1.978 (1.669-2.344)
HSI				
Q1 (<28.06)	1.000	1.000	1.000	1.000
Q2 (28.06-30.66)	1.377 (1.151-1.647)	1.354 (1.131-1.621)	1.226 (1.020-1.475)	1.220 (1.014-1.468)
Q3 (30.67-34.29)	1.926 (1.627-2.281)	1.858 (1.567-2.203)	1.999 (1.688-2.367)	1.957 (1.651-2.321)
Q4 (≥34.30)	4.088 (3.503-4.772)	3.808 (3.254-4.457)	3.833 (3.278-4.482)	3.602 (3.072-4.224)

^*^ Model 1 was crude model; Model 2 adjusted for gestational weeks of recruitment, maternal age, maternal BMI before pregnancy (except for HSI), FA supplement, first pregnancy, FBG and TC. Abbreviations: GH, gestational hypertension; PE, preeclampsia; OR, odds ratio; CI, confidence interval; AST, aspartate aminotransferase; ALT, alanine aminotransferase; GGT, γ-glutamyl transferase; HSI, hepatic steatosis index; Q1, quartile 1; Q2, quartile 2; Q3, quartile 3; Q4, quartile 4; BMI, body mass index; FA, folic acid; FBG, fasting blood glucose; TC, total cholesterol.

**Table 3:** Adjusted odds ratios for GH with the highest quantile of liver enzyme levels, stratified by baseline characteristics.

Subgroups	Number of subjects	AST		ALT		GGT		HSI	
		OR* (95%CI)	*P-*value^**^	OR* (95%CI)	*P*-value^**^	OR* (95%CI)	*P*-value^**^	OR* (95%CI)	*P*-value^**^
Maternal age, years									
< 35	28365	1.212 (1.029-1.427)	0.105	1.914 (1.603-2.284)	<0.001	2.054 (1.686-2.502)	0.039	4.502 (3.744-5.414)	<0.001
≥ 35	9161	0.942 (0.729-1.218)		1.054 (0.808-1.376)		1.427 (1.071-1.900)		2.351 (1.748-3.161)	
FBG in the first trimester, mmol/L									
< 5.6	37189	1.115 (0.971-1.281)	0.142	1.616 (1.394-1.873)	0.467	1.864 (1.584-2.194)	0.164	3.877 (3.312-4.538)	0.380
≥ 5.6	337	3.652 (0.758-17.590)		0.972 (0.246-3.862)		0.559 (0.101-3.089)		1.889 (0.397-8.996)	
Gravidity									
> 1	17337	1.133 (0.924-1.389)	0.991	1.542 (1.239-1.918)	0.654	1.752 (1.386-2.215)	0.584	3.721 (2.893-4.786)	0.825
1	20189	1.124 (0.930-1.357)		1.651 (1.352-2.015)		1.920 (1.532-2.407)		3.858 (3.152-4.721)	

^*^Adjusted for gestational weeks of recruitment, maternal age (excepted for subgroup analyses stratified by maternal age), maternal BMI before pregnancy (excepted for HSI), FA supplement, first pregnancy (excepted for subgroup analyses stratified by gravidity), FBG (excepted for subgroup analyses stratified by FBG) and TC. ^**^P-value is from the test for the difference between the two OR derived from subgroup analysis using z statistic. Abbreviations: GH, gestational hypertension; OR, odds ratio; CI, confidence interval; AST, aspartate aminotransferase; ALT, alanine aminotransferase; GGT, γ-glutamyl transferase; HSI, hepatic steatosis index; FBG, fasting blood glucose.

**Table 4:** Adjusted odds ratios for PE with the highest quantile of liver enzyme levels, stratified by baseline characteristics.

Subgroups	Number of subjects	AST		ALT		GGT		HSI	
		OR^*^ (95%CI)	*P*-value^**^	OR^*^ (95%CI)	*P*-value^**^	OR^*^ (95%CI)	*P*-value^**^	OR^*^ (95%CI)	*P*-value^**^
Maternal age, years									
< 35	28326	1.293 (1.092-1.532)	0.480	1.591 (1.333-1.898)	0.418	2.101 (1.712-2.579)	0.276	4.138 (3.435-4.985)	0.002
≥ 35	9120	1.157 (0.892-1.500)		1.387 (1.051-1.831)		1.707 (1.260-2.313)		2.341 (1.730-3.166)	
FBG in the first trimester, mmol/L									
< 5.6	37102	1.246 (1.080-1.437)	0.623	1.542 (1.328-1.792)	0.886	1.960 (1.652-2.325)	0.555	3.653 (3.115-4.284)	0.717
≥ 5.6	344	1.674 (0.477-5.872)		1.385 (0.360-5.332)		3.719 (0.452-30.599)		5.295 (0.676-41.451)	
Gravidity									
> 1	17247	1.370 (1.104-1.699)	0.304	1.592 (1.270-1.995)	0.720	2.197 (1.687-2.861)	0.306	3.831 (2.935-5.002)	0.552
1	20199	1.178 (0.975-1.422)		1.505 (1.233-1.836)		1.830 (1.465-2.287)		3.455 (2.830-4.219)	

^*^Adjusted for gestational weeks of recruitment, maternal age (excepted for subgroup analyses stratified by maternal age), maternal BMI before pregnancy (excepted for HSI), FA supplement, first pregnancy (excepted for subgroup analyses stratified by gravidity), FBG (excepted for subgroup analyses stratified by FBG) and TC. ^**^P-value is from the test for the difference between the two OR derived from subgroup analysis using z statistic. Abbreviations: PE, preeclampsia; OR, odds ratio; CI, confidence interval; AST, aspartate aminotransferase; ALT, alanine aminotransferase; GGT, γ-glutamyl transferase; HSI, hepatic steatosis index; FBG, fasting blood glucose.

**Table 5:** Area under curves (AUCs) to predict GH and PE by different liver enzyme indicators.

Indicators	GH		PE	
	AUC (95% CI)	*P*-value^**^	AUC (95% CI)	*P*-value^**^
AST	0.523 (0.508-0.537)^ *^	<0.001	0.532 (0.517-0.547)^ *^	<0.001
ALT	0.587 (0.573-0.601)^ *^	<0.001	0.585 (0.570-0.600)^ *^	<0.001
GGT	0.615 (0.601-0.629)^ *^	<0.001	0.612 (0.597-0.626)^ *^	<0.001
HSI	0.663 (0.649-0.676)^ *^	-	0.657 (0.643-0.671)^ *^	-

^*^ *P-*value <0.05 for AUC of each indicator for GH and PE. ^**^ P-value for comparison between AUC of HSI and other indices for GH and PE. Abbreviations: AUC, area under curve; GH, gestational hypertension; PE, preeclampsia; AST, aspartate aminotransferase; ALT, alanine aminotransferase; GGT, γ-glutamyl transferase; HSI, hepatic steatosis index.

## Abbreviations

PE: pre-eclampsia
GH: gestational hypertension
NAFLD: non-alcoholic fatty liver disease
HSI: hepatic steatosis index
BMI: body mass index
CBCS: China birth cohort study
FBG: fasting blood glucose
TG: triglyceride
TC: total cholesterol
LDL-C: low-density lipoprotein cholesterol
HDL-C: high-density lipoprotein cholesterol
AST: aspartate aminotransferase
ALT: alanine aminotransferase
GGT: γ-glutamyl transferase
ISSHP: International Society for the Study of Hypertension in Pregnancy
SD: standard deviation
ANOVA: one-way analysis of variance
ROC: receiver operating characteristic
AUC: area under curve
OR: odds ratio
CI: confidence interval
NO: nitric oxide
IL-6: Interleukin 6
TNF-α: Tumour necrosis factor α
CCL2: C-C motif ligand 2
