# Supplementary material for: Predictivity of Hepatic Steatosis Index for Gestational Hypertension and Preeclampsia: a Prospective Cohort Study
Source: Int J Med Sci. 2025 Jan 21;22(4):834–44. doi: 10.7150/ijms.104943 (PMC11843148; doi:10.7150/ijms.104943)
Supplement: Supplementary file 1 — Supplementary figure 1: Flow chart. [file ijmsv22p0834s1.pdf]

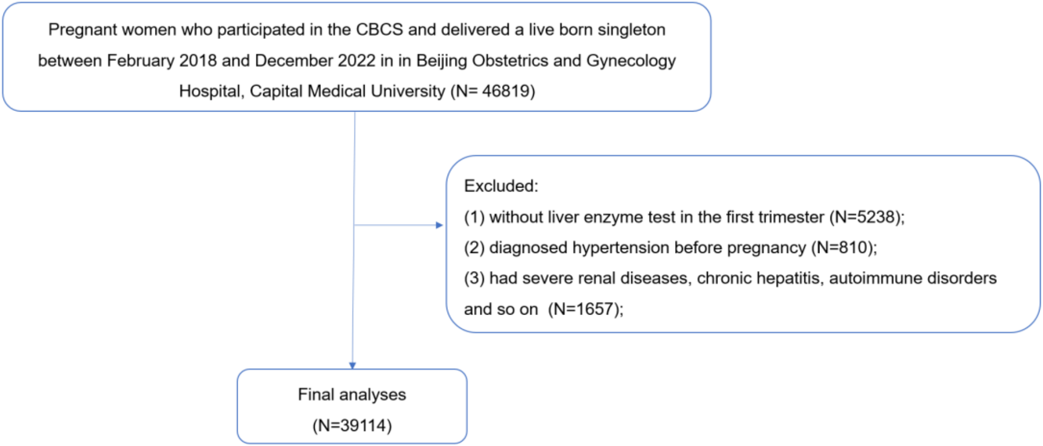
Pregnant women who participated in the CBCS and delivered a live born singleton between February 2018 and December 2022 in in Beijing Obstetrics and Gynecology Hospital, Capital Medical University (N= 46819)
Excluded:
(1) without liver enzyme test in the first trimester (N=5238);
(2) diagnosed hypertension before pregnancy (N=810);
(3) had severe renal diseases, chronic hepatitis, autoimmune disorders and so on (N=1657);
Final analyses
(N=39114)
